# Cystitis Cystica as a Large Solitary Bladder Cyst

**DOI:** 10.1089/cren.2017.0010

**Published:** 2017-03-01

**Authors:** Stephanie Potts, John Calleary

**Affiliations:** Pennine Acute Hospitals NHS Trust, North Manchester General Hospital, Manchester, United Kingdom.

**Keywords:** cystitis cystica, bladder cyst, lower urinary tract symptoms, suprapubic pain

## Abstract

***Background:*** In this case we describe the rare and not previously documented presentation of cystitis cystica as a large solitary cystic lesion within the bladder wall.

***Case Presentation:*** We present a case of a 46-year-old Russian male with a history of lower urinary tract symptoms and suprapubic pain. CT urogram showed a 5.8 cm filling defect/cystic mass related to the base of the bladder and prostate with 8 mm thick wall. The patient underwent cystoscopy and contrast study of bladder lesion with urethral dilatation and transurethral deroofing of bladder wall cyst under general anesthesia. A histologic diagnosis of cystitis cystica was made.

***Conclusion:*** This case described the rare presentation of a large solitary bladder cyst arising from the anterior bladder wall, identified histologically as cystitis cystica. Cystitis cystica presenting as a large cystic lesion of the bladder wall is rare; however, a diagnosis of cystitis cystica should be considered in unexplained cystic defects of the bladder wall.

## Introduction

Cystitis cystica is a rare chronic reactive inflammatory disorder thought to be caused by chronic irritation of the urothelium because of infection, calculi, outlet obstruction, or tumor^1^ resulting in multiple small filling defects in the bladder wall.

## Case Report

A 46-year-old male presented to urology as an outpatient with a history of lower urinary tract symptoms (LUTSs) and suprapubic pain. A community ultrasound scan of the urinary tract requested by the general practitioner identified a cystic lesion of the bladder.

**FIG. 1.**
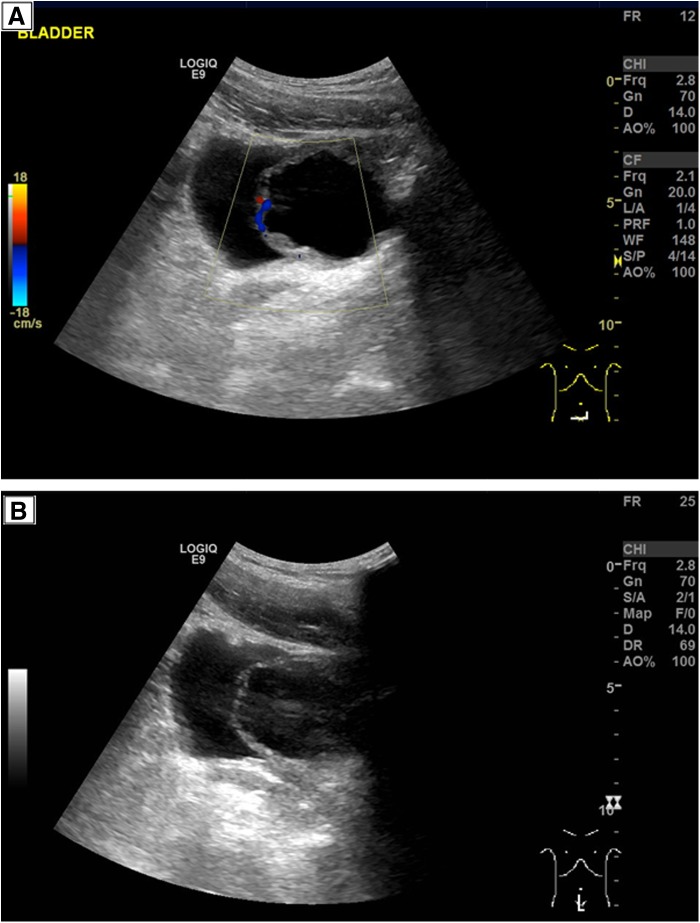
**(A, B)** Abdominal ultrasound scan showing the cystic lesion and thickened bladder wall.

The ultrasound scan identified a 5.9 × 5.4 × 5.9 cm irregularly shaped cystic lesion, with a thickened wall of 8.1 mm thought to be an ureterocele (Fig. 1).

On probing, he had a history of LUTSs with an international prostate symptom score of 9/35: frequency 2/5, urgency 1/5, nocturia 1/5, intermittency 1/5, straining 1/3, incomplete emptying 3/5 plus suprapubic and perineal pain and intermittent left testicular pain now known to be because of a radiologically identified left varicocele and episodes of prostatitis, likely chronic. Clinical examination was normal except for suprapubic tenderness. There was no history of frank hematuria or fever.

Urinalysis identified mild hematuria with small quantities of red blood cells, with no evidence of infection.

Prostate-specific antigen was within normal range at 0.423 ng/mL.

Initial diagnostic cystoscopy was performed; however, it proved to be inconclusive as the cystic lesion could not be observed and showed no obvious abnormality.

A contrast-enhanced CT urinary tract showed a 5.8 cm filling defect/apparent cystic mass related to the base of the bladder and prostate with 8 mm thick wall (usual bladder wall thickness >3 mm distended or >5 mm nondistended). Contents measure 48 HU before and after contrast. Contrast was noted to pool around the cystic mass on the delayed phase with the patient supine. Appearance was not typical of an ureterocele or transitional-cell carcinoma. Cystoscopy was recommended to exclude unusual ureterocele or cystic neoplasm.

**FIG. 2.**
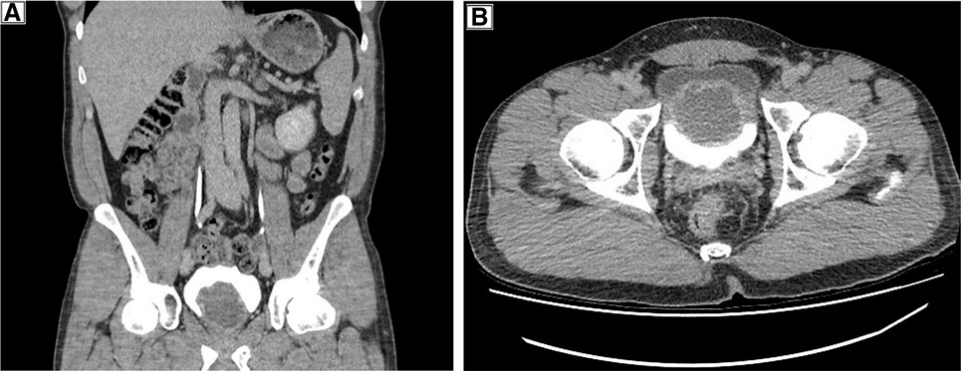
CT urogram showing the cystic bladder lesion. **(A)** Sagittal CT scan; **(B)** coronal cross-section showing the contrast-enhancing cystic bladder lesion.

Two stones at the lower groups of collecting system of left kidney 5 mm in size and a 1.3 cm simple left renal cyst were also reported (Fig. 2).

Local multidisciplinary team discussion recommended cystoscopy under general anesthesia to further examine the bladder lesion.

The various imaging modalities used identified this cystic lesion of the anterior wall of the bladder but not of the mucosa.

**FIG. 3.**
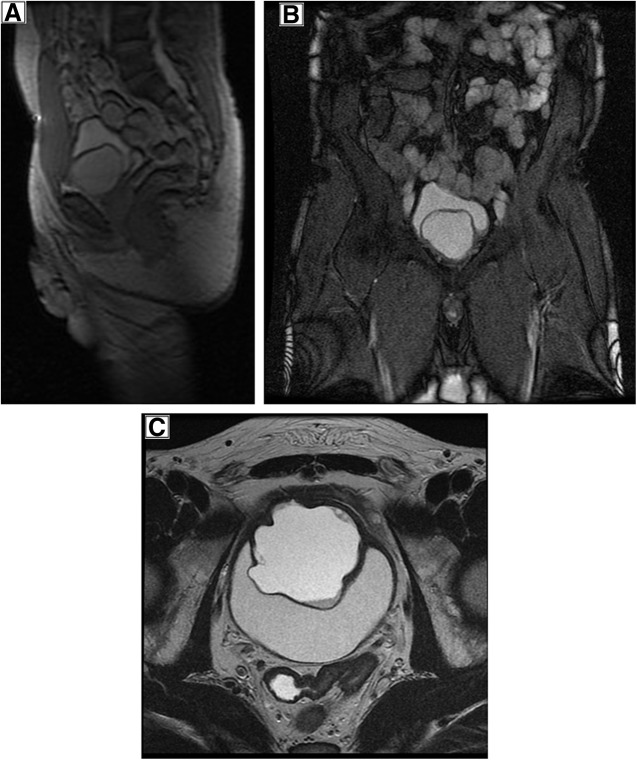
MRI pelvis/prostate showing the well-defined cystic lesion **(A)** 3pl LOC MRI; **(B)** Cor 2d Fiesta MRI; **(C)** T2 weighted ax FrFSE MRI scan.

**FIG. 4.**
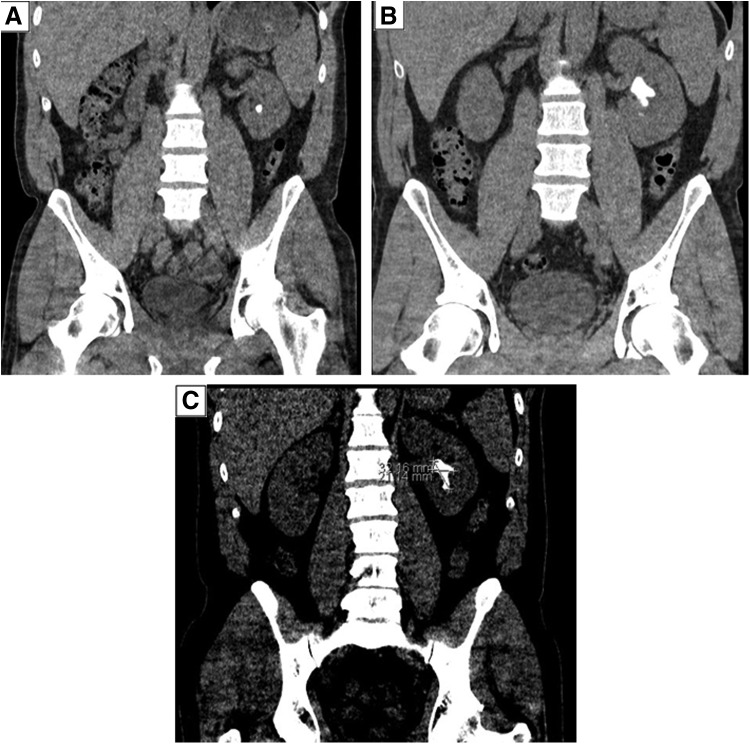
CT scan showing the progression of the left renal calculus from two 5 mm stones to a large staghorn calculus. **(A)** Noncontrast CT scan showing the small left lower pole renal calculus. **(B)** Noncontrast CT scan showing the 3.4 × 2 cm left staghorn calculus. **(C)** Noncontrast CT scan showing the remaining staghorn calculus after failed left ureteroscopy laser lithotripsy measuring 3.2 × 2.1 cm.

Contrast-enhanced MRI of pelvis and prostate describes a 6 × 5.5 cm intraluminal lesion in the bladder with asymmetric wall thickening, related to the bladder base, appearing separate to the prostate. The content of the lesion is of higher signal than normal bladder contents on T1W and T2W images (Figs. 3 and 4).

Eleven months after initial presentation to urology, the patient underwent cystoscopy and contrast study of the bladder lesion with urethral dilatation and transurethral deroofing of the bladder wall cyst (not urachal) under general anesthesia. On observation, the endothelium of the bladder appeared normal and the large cystic lesion arising from the anterior bladder wall was identified.

No obvious punctum was seen and within the cyst was an organized clot.

The lesion at the time of surgery appeared to be benign.

Biopsies were taken and sent for histologic analysis. A histologic diagnosis of cystitis cystica was made, with the overlying urothelium showing mild urothelial hyperplasia and no malignant change. Postoperatively, the patient experienced ongoing dull aching left flank pain and hematuria. However, the patient reports that his episodes of prostatitis had significantly improved. Although the patient's LUTSs were relatively mild (9/35), postoperatively the patient had a noticeable improvement in frequency, urgency, and incomplete emptying. Testicular pain remains an issue secondary to the currently untreated varicocele. Ten months before the initial bladder resection, two 5 mm nonobstructive lower pole stones were identified on CT urogram. Five months after initial resection, a CT urogram carried out identified a residual/recurrent discolored lesion seen in the bladder base along the posterior wall measuring ∼1.5 cm. A few small calcific densities within the bladder likely to represent small bladder calculi were also noted. The small calculus in the lower pole of the left kidney had now formed a staghorn calculus involving the middle and lower calices measuring 3.4 × 2 cm without causing any hydronephrosis or obstructive uropathy, given the low density (340–440 HU), which may represent a uric acid calculus.

Repeat flexible ureteroscopy identified discoloration in the region of the prostatic indentation. The patient underwent flexible ureteroscopy laser lithotripsy, Double-J stent insertion, and transurethral resection of residual bladder neck lesion.

Recent evaluation of the benign cystic lesion of bladder shows almost complete resolution.

The resected lesion from the bladder neck contained superficial fragments of diathermized and crushed detrusor muscle with a focal area of calcification; no surface urothelium or evidence of malignancy was identified.

The decision to use endoscopic laser lithotripsy through ureteroscopy was because of patient preference in an attempt to render him stone free in one treatment.

During the procedure, it was not possible to render the patient stone free and a considerable proportion of the staghorn calculus remained.

Two days post-Double-J stent insertion, the patient reattended with renal colic. Blood results were unremarkable, and an abdominal X-ray confirmed that the stent remained in the correct position. The ongoing pain was stent related and he was discharged with regular analgesia.

Postoperatively the patient had a degree of bleeding attributed to the resection of the bladder mucosa, and given that he has an indwelling stent, he may experience further episodes of visible hematuria.

As a result of the incomplete laser lithotripsy, a further noncontrast CT KUB 4 weeks after treatment identified that the staghorn calculus within the left kidney was similar to the previous scan. Although the calculus appears less dense compared with the previous CT, it appears almost similar in size and measures ∼3.2 × 2.1 cm. An incidental simple renal cyst in the upper pole of the left kidney remains unchanged. There is a suggestion of subtle irregularity in the bladder that has not been optimally assessed through scan, and correlation with repeat cystoscopy is recommended.

The patient's preference for further management would be for flexible ureteroscopy left laser lithotripsy, with the aim of rendering him stone free in that one sitting. Future scheduled intervention of repeat flexible ureteroscopy laser lithotripsy of the significant volume residual stone is planned at the patient's request and the patient will continue to be followed up as an outpatient with CT scans, ureteroscopy, and evaluation of ongoing clinical symptoms.

## Discussion

Cystitis cystica is a benign proliferative lesion of the bladder as a result of a chronic reactive inflammatory disorder thought to be caused by chronic irritation of the urothelium because of infection, calculi, obstruction, or tumor.^1,2^

Cystitis cystica is characterized by hyperplasia of the bladder mucosa and the presence of cystically dilated von Brunn nests within the lamina propria. Cystitis cystica predominantly occurs at the bladder neck and trigone region of the bladder.

Minor forms are common and have similar clinical features as cystitis, but its major form may be mistaken for bladder tumor on cystoscopy.

CT imaging in cystitis cystica classically shows the appearance of multiple small filling defects of 2 to 5 mm in the bladder wall; however, in this case, cystitis cystica presented as a large solitary cystic lesion within the bladder wall not previously described in the literature.

Cystoscopic biopsy is mandatory. Submucosal nests of columnar epithelial cells surrounding a central liquefied region of columnar degeneration are histologically diagnostic.

Hematuria or symptoms of urinary tract infection are the usual presenting feature of cystitis cystic; however, patients can be asymptomatic.^3,4^

Cystitis cystica may occur at any age, and there is a slight male predominance.^1,2^ There is debate among specialists of the precancerous potential of cystitis cystica. Harik and O'Toole 2012^2^ considered cystitis cystica to be a premalignant lesion of the urinary tract, leading to adenocarcinoma of the bladder, although there is limited documentation of the progression of cystitis cystica to carcinoma of the bladder.

This patient has a positive family history of bladder cancer, with his father previously diagnosed with the condition. However, the prevalence of cystitis cystica has been reported at 60% in normal bladders on autopsy and Wiener et al.^4^ suggested that cystitis cystica may be a normal urothelial variant with no precancerous potential.

## Conclusion

This case described the rare presentation of a large solitary bladder cyst arising from the anterior bladder wall, identified histologically as cystitis cystica.

Cystitis cystica presenting as a large cystic lesion of the bladder wall is rare; however, a diagnosis of cystitis cystica should be considered in unexplained cystic defects of the bladder wall.

## Abbreviations Used

CT: computed tomography
HU: Hounsfield units
KUB: kidney, ureter, and bladder radiograph
LUTSs: lower urinary tract symptoms
MRI: magnetic resonance imaging
